# Simultaneous use of traditional Chinese medicine (Si-Ni-Tang) to treat septic shock patients: study protocol for a randomized controlled trial

**DOI:** 10.1186/1745-6215-12-199

**Published:** 2011-08-24

**Authors:** Huang-Chi Chen, Wen-Chi Chen, Kai-Huang Lin, Yung-Hsiang Chen, Lun-Chien Lo, Tsung-Chieh Lee, Te-Chun Hsia, Chu-Hsien Wang, Shin-Hwar Wu, Hsin-Whae Hsu, Yu-Jun Chang, Yu-Chuen Huang, Tien-Hsiung Ku, Ming-Hwarng Horng

**Affiliations:** 1 Graduate Institute of Integrated Medicine, College of Chinese Medicine, China Medical University, Taichung, Taiwan; 2 Division of Critical Care Medicine, Department of Internal Medicine, Changhua Christian Hospital, Changhua, Taiwan; 3 Department of Chinese Medicine, Changhua Christian Hospital, Changhua, Taiwan; 4 Laboratory of Epidemiology and Biostatistics, Changhua Christian Hospital, Changhua, Taiwan; 5 School of Chinese Medicine, College of Chinese Medicine, China Medical University, Taichung, Taiwan; 6 Department of Anesthesiology, Changhua Christian Hospital, Changhua, Taiwan; 7 Graduate Institute of Chinese Medicine, College of Chinese Medicine, China Medical University, Taichung, Taiwan

## Abstract

**Background:**

Even though there are continually upgraded recommendations for managing sepsis, such as "Surviving Sepsis Campaign: international guidelines for management of severe sepsis and septic shock", mortality is still high. Si-Ni-Tang, a remedy documented in Shanghan Lun, a medical collection from ancient China, is used for treating patients with sepsis and septic shock. Using a well-designed clinical trial, we are eager to survey the effectiveness of the concurrent use of this remedy in restoring these patients' hemodynamic status, or "Yang Qi".

**Methods/Design:**

Patients admitted to our medical intensive care units with the diagnosis of septic shock, defined as persistent hypotension induced by sepsis despite adequate fluid resuscitation, are eligible for participation. The inclusion criteria include: age from 20 to 85 years, conditions meeting the definition of septic shock, use of vasopressors within 24 hours of entering the study, and use of a nasogastric tube for feeding. The enrolled patients are randomly allocated either to the Si-Ni-Tang group or the placebo group. The prescription of the trial drugs (Si-Ni-Tang/placebo) is 2.25 grams 4 times a day for 7 days or till shock reversal (if shock reversal occurs in less than 7 days). Data, including duration of vasopressor infusion, gender, age, co-morbidities, APACHE II score, predicted mortality, ICU mortality, ICU length of stay, hospital mortality, hospital length of stay, source of sepsis, and culture results, are collected for the following analysis.

**Discussion:**

Si-Ni-Tang is composed of processed *Zingiber officinale*, *Glycyrrhiza uralensis*, and *Aconitum carmichaeli*. *Zingiber officinale *and *Glycyrrhiza uralensis *are found to have the ability to reduce pro-inflammatory cytokine production, to inhibit lipopolisaccharide-induced macrophage activation and function, and to lessen the bacterial load and suppress acute and chronic inflammation. *Aconitum carmichaeli *is known to have vasopressor activity, and positive chronotropic and inotropic effects. As this remedy has a potential benefit in treating septic shock patients, we designed a double-blind, prospective, randomized controlled trial and would like to publish the results and conclusions later.

**Trial Registration:**

ClinicalTrials.gov: NCT01223430

## Background

Sepsis is an infectious disease, usually caused by bacterial infection, with systemic manifestations. Severe sepsis is defined as sepsis with organ dysfunction or tissue hypoperfusion related to sepsis. If the disease progresses to persistent hypotension, even with adequate fluid resuscitation, the presentation is known as septic shock.

Severe sepsis, and if further deteriorated, septic shock, are important issues for healthcare workers. Even though the recommendations for managing sepsis and septic shock, such as "Surviving Sepsis Campaign: international guidelines for management of severe sepsis and septic shock"[1], are continually upgraded, mortality due to sepsis and septic shock in the intensive care units is still high.

We may not know initially the exact etiology of the septic shock when treating the patients, but maintaining an adequate hemodynamic status in order to keep these patients with organ perfusion well is always the main issue for intensivists.

The history of Chinese medicine can be traced back thousands of years. Certainly, the theory and application of Chinese medicine in treating patients with severe infection have been documented in several Chinese medical books. The presentation of the septic shock patient is characterized as "depletion of Yang Qi", meaning little energy and very weak pulse.

When treating patients diagnosed with septic shock, the optimal approach we will take will be to follow recently proposed guidelines. However, there is still much research devoted to finding a better way to improve patient outcome. Herbal or Chinese medicine has been investigated in the treatment of infectious disease and shock[2]. Many remedies to treat sepsis or septic shock have been documented in ancient Chinese medical books or literature. Si-Ni-Tang, for example, documented in Shanghan Lun, a medical collection from ancient China, is used for treating patients with shock, heart failure, severe watery diarrhea and poor extremity circulation. It is considered a good choice to treat patients who suffer from infectious disease complicated with hemodynamic instability.

Si-Ni-Tang is manufactured from and composed of processed *Zingiber officinale*, *Glycyrrhiza uralensis*, and *Aconitum carmichaeli*. Using a well-designed clinical trial, we are eager to survey the effectiveness of concurrent use of this remedy in restoring "Yang Qi". That is to say, we want to know whether Si-Ni-Tang can shorten the duration of the shock status. The purpose of this study is to determine whether the simultaneous use of a traditional Chinese medicine, Si-Ni-Tang, is more effective in the treatment of septic shock patients. Only by conscientiously performing studies and pursuing positive outcomes can we push Chinese medicine onto the stage. Certainly, finding the best way to benefit people and patients with septic shock is the ultimate goal of our study.

## Methods/Design

The study is designed as a double-blind, prospective, randomized controlled trial. It has been approved by the ethics committee. Our study procedures and informed consent were reviewed by the Institutional Review Board of Changhua Christian Hospital beginning in Jan. 2010. There were four revisions before the study procedures and informed consents were finally approved by the third Institutional Review Board Committee on Aug. 10, 2010.

All participating patients are randomized using a computerized block randomization schedule with a multiple block size of four. 120 patients (30 blocks) were randomly (same chance) generated that were kept in sequentially numbered opaque envelopes. Researchers and patients are blinded to treatment allocation; the only un-blinded individual is the statistician responsible for randomization process. The treatment assignments are balanced within each block. Each block contains 4 patients with 2 patients for Si-Ni-Tang group, and 2 patients for placebo group (same chance).

Our study is directed by the Graduate Institute of Integrated Medicine, College of Chinese Medicine, China Medical University and is conducted in the medical intensive care units in Changhua Christian Hospital, a medical center in central Taiwan. The drugs used in the trial, whether Si-Ni-Tang or placebo, are both manufactured and provided by a pharmaceutical company that meet the requirements of Good Manufacturing Practice. All important drug information, including ingredient composition, heavy metals, etc., is provided by the same company.

According to the GUIDELINE ON CLINICAL TRIAL OF NEW CHINESE MEDICINE promulgated by the Department of Health, Executive Yuan, Taiwan on Feb. 5, 2008, we are allowed to investigate directly the therapeutic efficacy of the Chinese medicines listed in classical Chinese pharmacopoeias, due to the long-term experience and widespread clinical use of these medicines. The drug used in our trial, Si-Ni-Tang, an example of Chinese medicines listed in classical Chinese pharmacopoeias, is regarded as safe and effective for patients with shock. However, it has been criticized for lacking adequate evaluation through clinical trials supported and conducted according to the principles of modern clinical trials. To evaluate the actual benefit and the possible risk, we designed this double-blind, prospective, randomized controlled trial.

Our study is being carried out somewhat like phase 2 studies in clinical trials. Phase 2 studies are set forth to evaluate the effectiveness of drugs in participants with a certain disease or condition. We assume that the mean duration of vasopressor use of the Si-Ni-Tang group is 3 hours shorter than that of the placebo group, with a standard deviation of 6.5 hours. Under the setting of alpha = .05 and power = 80%, the calculated sample size of each group is around 60. This is why we plan to enroll 120 participants.

**Figure 1:**
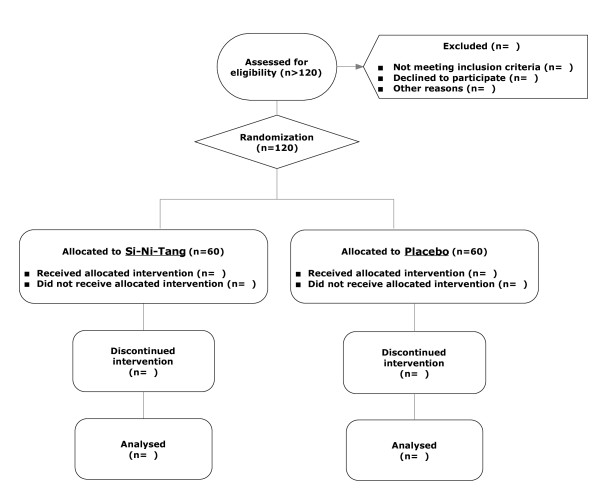
**Enrolment Flowchart**. The figure illustrates how the participants are enrolled and allocated.

Sepsis is an infectious disease, usually caused by bacterial infection, with systemic manifestations. Sepsis-induced hypotension is defined as a systolic blood pressure less than 90 mmHg or mean arterial pressure less than 70 mmHg. Septic shock is defined as persistent hypotension induced by sepsis, despite adequate fluid resuscitation. We plan to enroll 120 patients who have been admitted to our medical intensive care units with the diagnosis of septic shock. The flow chart of the study is illustrated as Figure 1.

Eligible patients must fulfill all the following requirements: age from 20 to 85 years, a disease diagnosis meeting the definition of septic shock, use of vasopressors now and an initiation of vasopressor use within 24 hours of entering the study, use of a nasogastric tube for feeding, and informed consent obtained from either the patients or their relatives.

Exclusion criteria are an extremely poor gastrointestinal function and an inability to tolerate diet feeding, acute myocardial infarction, evidence of major bleeding, expected or scheduled surgical intervention in the subsequent one week, use of 2 or more than 2 kinds of vasopressors upon entrance into the study, and patients who have received digoxin for arrhythmia within the past one week.

The treatment for the patients with septic shock in our hospital adheres to the Surviving Sepsis Campaign: International guidelines for management of severe sepsis and septic shock 2008. Our study uses block randomization with a block size of 4 and an allocation ratio of 1:1. After enrolment, the eligible patients are either allocated to the Si-Ni-Tang group or the placebo group. The prescription of the trial drugs is 2.25 grams 4 times a day (9 grams per day) for 7 days or till shock reversal (if shock reversal occurs in less than 7 days). Shock reversal is defined as the discontinuation of norepinephrine or dopamine for at least 24 hours.

We will collect the following data from each participant: gender, age, co-morbidities, APACHE II score, predicted mortality, ICU mortality, ICU length of stay, hospital mortality, hospital length of stay, source of sepsis (pulmonary, gastrointestinal, renal or others), culture results (Gram-positive, Gram-negative or mixed) and duration of vasopressor infusion. Shock reversal and time to cessation of vasopressor use would be the primary outcome measurements.

Baseline variables will be evaluated for balance between the two groups using the Student's t-test for unpaired data for the comparison of continuous variables and the Pearson's chi-squared test to compare categorical variables.

The effect of Si-Ni-Tang treatment on time to cessation of vasopressor use is estimated, first, from Kaplan-Meier curves and log-rank test for median time, and second, from adjusted Cox proportional hazards regression models for potential confounders (age, APACHE II score at baseline, source of sepsis, co-morbidities). Corresponding hazard ratios along with their 95% confidence intervals are reported. Patients who died before cessation of vasopressor use are treated as censored. Analysis is performed on the intention to treat population.

There is no planned subgroup analysis anticipated. Only primary and secondary outcome measures will be analyzed. However, all of the variables which are found to be important predictors of shock reversal and adverse outcome will be used for stratifications for post-hoc analysis (relative risks will be estimated for the major outcomes using multivariate Poisson regression adjusting for covariates).

## Discussion

Septic shock, usually caused by bacterial infection, is a severe systemic inflammatory consequence leading to multi-organ dysfunction. As with acute myocardial infarction, stroke and multiple trauma, treatment guidance for severe sepsis and septic shock in the initial period has been developed. Even though treatment recommendations are continually upgraded, mortality from sepsis and septic shock in the intensive care units is still high. Many remedies using herbal or traditional Chinese medicine have been proven to be potential adjuvant therapies in the treatment of sepsis and septic shock.

Si-Ni-Tang is composed of processed *Zingiber officinale*, *Glycyrrhiza uralensis*, and *Aconitum carmichaeli*. *Zingiber officinale *extract is found to have the ability to inhibit pro-inflammatory cytokines[3] and lipopolisaccharide-induced macrophage activation and function[4], and to reduce the bacterial load and suppress acute and chronic inflammation[5]. *Glycyrrhiza uralensis *extract is also considered to have anti-inflammatory properties and reduce lipopolysaccharide-induced pro-inflammatory cytokine secretion[6-8]. "Fuzi", the lateral root tuber of processed *Aconitum carmichaeli*, is used for treating pain, arthritis, cardiogenic shock, rheumatism, etc., in traditional Chinese medicine. Aconite roots are used only after processing and the procedure can hydrolyze aconite alkaloids into less toxic and non-toxic derivatives[9]. Aconite roots may contain coryneine chloride and higenamine, and possess vasopressor activity and positive chronotropic and inotropic effects[10,11]. Without a doubt, "Fuzi", *Aconitum carmichaeli*, is the critical factor in the remedy, Si-Ni-Tang.

Each component of Si-Ni-Tang is considered to be beneficial to septic shock patients, as described above. However, the most important theory of the Chinese medicine prescription is that there is a leading, coordinating, auxiliary and guiding effect in the remedy component. Perhaps, the exact benefit and effect of the drug combination go beyond what we already know. Only via conscientiously performed studies and positive outcomes can we push Chinese medicine onto the stage. And again, benefiting people and patients with septic shock is always the ultimate goal of our research. The clinical trial is being conducted now. If there are any conclusions in the near future, we would like to publish them later.

## List of abbreviations

APACHE II: Acute Physiology and Chronic Health Evaluation II; ICU: Intensive Care Unit.

## Competing interests

The authors declare that they have no competing interests.

## Authors' contributions

All the authors read and approved the final manuscript. HCC and MHH have full access to all of the data in the study and take responsibility for the integrity of the data and the accuracy of the following data analysis. *Study concept and design: *HCC, MHH, KHL, WCC, YHC, TCH, LCL. *Drafting of the manuscript: *HCC, MHH. *Critical revision of the manuscript for important intellectual content: *WCC, KHL. *Acquisition of data*: HCC, TCL, CHW, SHW, HWH, THK. *Randomization design and statistical analysis of the collected data*: YJC, YCH. *Obtained funding: *HCC, MHH, KHL.
